# Bioactive components and potential mechanisms of Biqi Capsule in the treatment of osteoarthritis: based on chondroprotective and anti-inflammatory activity

**DOI:** 10.3389/fphar.2024.1347970

**Published:** 2024-04-17

**Authors:** Ziyue Jia, Jiale Zhang, Xintong Yang, Huiyou Chen, Yuxing Wang, Opoku Bonsu Francis, Yuanchao Li, Zhanbiao Liu, Shaozhuo Zhang, Qilong Wang

**Affiliations:** ^1^ School of Chinese Materia Medica, Tianjin University of Traditional Chinese Medicine, Tianjin, China; ^2^ Institute of Traditional Chinese Medicine, Tianjin University of Traditional Chinese Medicine, Tianjin, China; ^3^ State Key Laboratory of Component-based Chinese Medicine, Tianjin University of Traditional Chinese Medicine, Tianjin, China

**Keywords:** osteoarthritis, Biqi capsule, PI3K/Akt/mTOR pathway, NF-κB/IL-6 pathway, bioactive component

## Abstract

Cartilage damage and synovial inflammation are vital pathological changes in osteoarthritis (OA). Biqi Capsule, a traditional Chinese medicine formula used for the clinical treatment of arthritis in China, yields advantages in attenuating OA progression. The drawback here is that the bioactive components and pharmacological mechanisms by which Biqi Capsule exerts its anti-inflammatory and chondroprotective effects have yet to be fully clarified. For *in vivo* studies, a papain-induced OA rat model was established to explore the pharmacological effects and potential mechanisms of Biqi Capsule against OA. Biqi Capsule alleviated articular cartilage degeneration and chondrocyte damage in OA rats and inhibited the phosphorylation of NF-κB and the expression of pro-inflammatory cytokines in synovial tissue. Network pharmacology analysis suggested that the primary biological processes regulated by Biqi Capsule are inflammation and oxidative stress, and the critical pathway regulated is the PI3K/AKT signaling pathway. The result of this analysis was later verified on SW1353 cells. The *in vitro* studies demonstrated that Glycyrrhizic Acid and Liquiritin in Biqi Capsule attenuated H_2_O_2_-stimulated SW1353 chondrocyte damage via activation of PI3K/AKT/mTOR pathway. Moreover, Biqi Capsule alleviated inflammatory responses in LPS-stimulated RAW264.7 macrophages via the NF-κB/IL-6 pathway. These observations were suggested to have been facilitated by Brucine, Liquiritin, Salvianolic Acid B, Glycyrrhizic Acid, Cryptotanshinone, and Tanshinone ⅡA. Put together, this study partially clarifies the pharmacological mechanisms and the bioactive components of Biqi capsules against OA and suggests that it is a promising therapeutic option for the treatment of OA. Chemical compounds studied in this article. Strychnine (Pubchem CID:441071); Brucine (Pubchem CID:442021); Liquiritin (Pubchem CID:503737); Salvianolic Acid B (Pubchem CID:6451084); Glycyrrhizic Acid (Pubchem CID:14982); Cryptotanshinone (Pubchem CID:160254); Tanshinone ⅡA (Pubchem CID:164676).

## Highlights

• Biqi Capsule ameliorates papain-induced cartilage damage and inflammatory response.
• Biqi Capsule exerts its chondroprotective effect by activating the PI3K/AKT/mTOR pathway in chondrocytes and an anti-inflammatory effect by inhibiting the NF-κB/IL-6 pathway.
• Glycyrrhizic Acid and Liquiritin alleviate chondrocyte damage via the PI3K/AKT/mTOR pathway.

## 1 Introduction

Osteoarthritis is a complex disease characterized by pathological changes across all joint tissues, including cartilage, subchondral bone, ligaments, menisci, joint capsule, and synovium (Loeser et al., 2016). The incidence of OA is increasing globally, making it one of the primary diseases that reduce the quality of life and can even lead to disability in older adults (Allen et al., 2022). Currently, an estimated 240 million individuals worldwide have symptomatic OA. Low-grade chronic inflammation of the synovium plays a central role in the pathological progression of OA (Coaccioli et al., 2022). Pro-inflammatory mediators, such as cytokines, lipid mediators, and reactive oxygen species (ROS), produced by synoviocytes are responsible for the degradation of the extracellular matrix and cartilage loss (Zahan et al., 2020). In addition, the mediators that cause cartilage damage in OA (such as damage-associated molecular patterns and extracellular matrix components) can exude into the synovial fluid and recruit synovial macrophages, which further produce an excess of pro-inflammatory cytokines, accelerating cartilage degradation (Coaccioli et al., 2022). In summary, the triad of cartilage destruction, synovial inflammation, and macrophage activation contributes to an ongoing sterile wound-healing “vicious circle” resulting in joint tissue pathology. Modulating these cellular processes or intervening with factors that modify the phenotypic state of the cells may be a promising approach to slowing down OA development (Kulkarni et al., 2021). Biqi Capsule (NMPA approval number: Z10910026) is a Chinese traditional patent medicine used for the treatment of OA (SPGD, 2021). Biqi Capsule is composed of 10 Chinese herbs, including *Strychnos nux-vomica* L., *Pheretima aspergillum* (E. Perrier), *Codonopsis pilosula* (Franch.) Nannf., *Poria cocos* (Schw.) Wolf., *Atractylodes macrocephala* Koidz., *Ligusticum chuanxiong* Hort., *Salvia miltiorrhiza* Bunge, *Panax notoginseng* (Burk.) F. H. Chen ex C. Chow, *Achyranthes bidentata* BL., and *Glycyrrhiza uralensis* Fisch. Previous pharmacological studies have shown that Biqi Capsule alleviates local joint and systemic inflammation, synovial hyperplasia, and cartilage destruction caused by collagen-induced arthritis (CIA) (Wang et al., 2018), and its anti-inflammatory effect may be a result of inhibition of the NO-iNOS pathway and COX-2 pathway (Wang et al., 2011). The chemical components of Biqi Capsule have already been identified according to previous studies (Wang et al., 2011; Jing et al., 2018; Xing-Yan et al., 2021). However, the pharmacological mechanisms and bioactive components of Biqi Capsule which facilitate the treatment of OA remain largely unexplored.

In this study, we evaluate the anti-inflammatory and chondroprotective effects of Biqi Capsule using a papain-induced rat model of OA. Network pharmacology analysis and pathway-blocking experiments were used to explore the underlying mechanisms. Molecular docking and screening based on H_2_O_2_-stimulated SW1353 cells and LPS-stimulated RAW264.7 cells were performed to better assess the molecular dynamics and pharmacological activities of the bioactive components. Our results revealed the underlying mechanisms and bioactive components of Biqi Capsule in treating OA.

## 2 Materials and methods

### 2.1 Reagents and antibodies

Biqi Capsules were provided by Tianjin Darentang Pharmaceutical Jingwanhong Co. Ltd. (Tianjin, China) with the production batch number 31174066. The quality of Biqi Capsule is supervised by the National Medical Products Administration (NMPA) according to the Pharmacopoeia of the People’s Republic of China 2020 Edition, Part I, Page 1808. Papain (76,220), poloxamer (P4894), Lipopolysaccharide (LPS, L2880), and Dimethyl sulfoxide (DMSO, D8418) were purchased from Sigma-Aldrich (Shanghai, China). LY294002 was purchased from MedChemExpress (NJ, USA). Strychnine, Brucine, Liquiritin, Salvianolic Acid B, Glycyrrhizic Acid, Cryptotanshinone, and Tanshinone ⅡA were purchased from Sichuan Vicky Biotechnology Co., Ltd. (Chengdu, China). The primary antibodies against PI3K p85 (#4257), Akt (#9272), p-Akt (#9275), NF-κB p65 (#8242), p-NF-κB p65 (#3033), PCNA (#13110), the second antibodies against rabbit (#7074) and mouse (#7076) IgG were purchased from Cell Signaling Technology (MA, USA). The primary antibodies against p-PI3K p85 (ab182651) and β-actin (ab8226) were purchased from Abcam (Cambridge, UK). The primary antibodies against mTOR (A11355) and p-mTOR (AP0978) were purchased from ABclonal Technology (Wuhan, China). Rat PGE2 Elisa Kit (LCSJZF30697) was purchased from LunChangShuo Biotech. (Xiamen, China); Rat IL-6 Elisa Kit (E04640r) was purchased from CUSABIO (Wuhan, China); Rat TNF-α Elisa Kit (KRC3011), Rat IL-1β Elisa Kit (BMS630) and mouse IL-6 ELISA Kit (88-7064-88) were purchased from Invitrogen (CA, USA).

### 2.2 Animals and models

**FIGURE 1:**
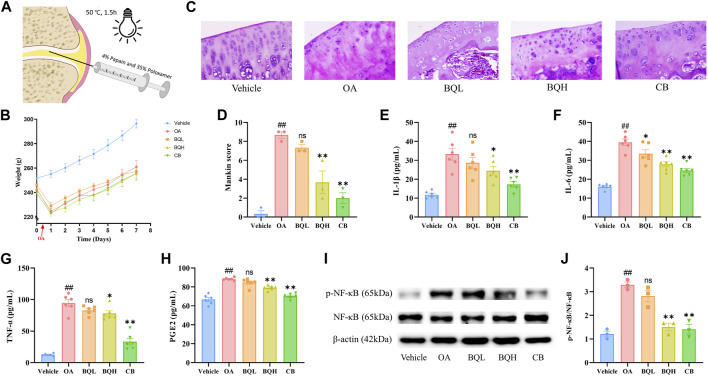
The efficacy of Biqi Capsule on papain-induced OA rats. The papain-induced OA rats were treated with Biqi Capsule for 7 days. **(A)** Construction of an OA rat model. **(B)** Weight of rats during the experiment. Data were present as mean ± SEM (n = 10). **(C)** Representative sections of HE staining knee joints from rats in different groups (400×). **(D)** HE staining sections of rat knee joints from each group were used for the assessment via Mankin score. Data were presented as mean ± SEM (n = 3, #*p* < 0.05, ##*p* < 0.01 compared with the Vehicle group; *p < 0.05, **p < 0.01 compared with the OA group). **(E–H)** After 7 days of treatment, the IL-1β, IL-6, TNF-α, and PGE2 levels in synovial tissue were examined by ELISA. Data were presented as mean ± SEM. (n = 6, #*p* < 0.05, ##*p* < 0.01 compared with the Vehicle group; *p < 0.05, **p < 0.01 compared with the OA group). **(I, J)** After 7 days of treatment, the phosphorylation of NF-κB (p65) in the synovial tissue was examined by Western Blot. The relative expression was quantified by ImageJ software. Data were presented as mean ± SEM. (n = 3, #*p* < 0.05, ##*p* < 0.01 compared with the Vehicle group; *p < 0.05, **p < 0.01 compared with the OA group).

Healthy male SD rats (n = 60, body weight: 160g–180 g) were purchased from SPF Biotechnology Co., Ltd. (Beijing, China). All experimental procedures were approved by the Animal Care and Use Committee of the Tianjin University of Traditional Chinese Medicine (Authorization number: TCM-LAEC2022129). All animals were kept at 22°C–26 °C and 55% ± 5% humidity with a 12 h light/dark cycle and allowed free access to food and water during the experiments. After a week of adjustment, all of the rats were randomly divided into five groups as follows: Vehicle group (equal normal saline solution, i. g.), OA group (equal normal saline solution, i. g.), BQL group (Biqi Capsule low dose, 0.648 g/kg·d, i. g., double clinical dose), BQH group (Biqi Capsule high dose, 1.296 g/kg·d, i. g.), Celecoxib group (CB, as a positive control, 24 mg/kg·d, i. g.). The rat model of OA was established using the invention patent method of the author’s research group, which has been authorized by the China National Intellectual Property Administration (Approval number: CN102058877A). Previous studies have demonstrated that this model successfully simulates the pathophysiological processes of OA in humans and is suitable for pharmacological studies and efficacy evaluation of anti-osteoarthritic drugs (Zhanbiao et al., 2024). Briefly, the model of OA was induced by a sustained-release papain agent (4% papain and poloxamer). The sustained-release papain agent was injected into both knee joint compartments of rats anesthetized with pentobarbital and then monitored for 1.5 h at 50°C (Figure 1A).

### 2.3 Biqi Capsule experimental design

For the *in vivo* study, rats had undergone a single intra-articular injection with 50μL/knee joint sustained-release papain agent. The day after the establishment of the OA model, BQL and BQH administration groups received the indicated dosage of Biqi Capsule content powder (dissolved in double distilled water) by oral gavage daily for seven consecutive days. Meanwhile, rats in the OA and CB groups were administered water and celecoxib, respectively. The dose was administered as described above.

For the *in vitro* study, a solution from Biqi Capsule extract was prepared as follows (Wang et al., 2011): 10 g of Biqi Capsule content powder was mixed with 100 mL double-distilled water, extracted by ultrasonication twice at 37 °C for 1 h each. The extraction solution was centrifuged at 12,000rpm for 30 min and then freeze-dried to obtain the aqueous extract of Biqi Capsule. The extract was dissolved in a medium before use and filtered through a 0.22 µm pore size membrane filter (Millipore, USA).

### 2.4 Keen joint hematoxylin and eosin staining

Rats were euthanized on the eighth day to obtain the knee joints. The knee joints were fixed in 4% paraformaldehyde solution, decalcified with 1% nitric acid for 48h, dehydrated in gradient ethanol and xylene, and embedded in paraffin wax. Knee joint sections (4 μm) were stained with hematoxylin and eosin (HE) staining, and the cartilage abnormalities were evaluated by the modified Mankin score (Mankin and Lippiello, 1970; Mankin et al., 1971; Mankin et al., 1981; Takahashi et al., 2019; Sun et al., 2020).

### 2.5 Detection of inflammatory factors in tissue homogenate

Synovial tissues were rinsed with pre-chilled PBS, the blood was removed, and the tissues were dried with filter paper. The synovial tissues were weighed accurately and ground in a homogenizer (Servicebio, Wuhan, China) by adding 0.1 mg/μL PBS. The prepared tissue homogenate was centrifuged at 4 °C and 12,000 rpm for 15 min, and the supernatant was retained. The levels of PGE2, IL-6, IL-1β, and TNF-α were determined by ELISA according to the manufacturer’s instructions.

### 2.6 Western blot analysis

Total protein from synovial tissue/cells was collected with RIPA lysis containing Phosphatase Inhibitor Cocktail and Protease Inhibitor Cocktail (Yeasen), then ground in a homogenizer. Concentrations of protein were determined by the BCA Protein Assay Reagent Kit. 10 μg protein was separated by SDS-PAGE electrophoresis and transferred to PVDF membranes (Merck, NJ, USA). After blocking with 5% fresh nonfat milk in tris-buffered saline containing 0.05% Tween-20, the membranes were incubated with primary antibody overnight at 4 °C, followed by HRP-conjugated secondary antibody incubation. The membranes were incubated with Chemiluminescent HRP Substrate (Merck) for 1 min, then images were developed through Amersham Imager 600 (GE, MA, USA). The relative expression was quantified by ImageJ software.

### 2.7 Network pharmacology analysis

#### 2.7.1 Target prediction of compounds in Biqi Capsule

Quality markers also known as “Q-markers” of Traditional Chinese Medicine are efficacy-associated markers, identified by integrating multidiscipline-based strategies (including natural products chemistry, analytical chemistry, bionics, computer-aided design, pharmacology, system biology, and pharmacodynamics) (Yang et al., 2017). It is a parameter used to assess the functional properties that exist in the raw materials and products of TCM (including the decoction materials, decoctions, extractives, and Chinese patent medicines), which can be used as the indicators for quality control of TCM to embody the effectiveness. Based on the identification of chemical constituents and the analysis of Quality-markers, seven Quality-markers of Biqi Capsule are summarized in Figure 3A (Wang et al., 2011; Jing et al., 2018; Xing-Yan et al., 2021).

The structure files (Mol2 format) of the compounds were downloaded from PubChem (https://pubchem.ncbi.nlm.nih.gov/), and uploaded to the PharmMapper server (https://lilab-ecust.cn/pharmmapper/index.html). The species is restricted to “human” for virtual screening of target prediction based on reverse molecular docking. The target prediction results were used to select the top 50% of the Fit value as the critical targets for the compounds and were imported into the UniProt database for their corresponding gene names.

#### 2.7.2 OA target gene prediction

OA-related targets were collected from three databases using “Osteoarthritis” as the keyword: the GeneCards database (https://www.genecards.org/), the Online Mendelian Inheritance in Man (OMIM) database (https://www.omim.org/) and the Therapeutic Target Database (TTD) (http://db.idrblab.net/ttd).

#### 2.7.3 GO and KEGG analysis

The BQ targets identified in Section 2.7.1, along with the OA targets identified in Section 2.7.2, were both uploaded to the Draw Veen Diagram website (https://bioinformatics.psb.ugent.be/webtools/Venn/). Overlapping targets in the Veen diagram were taken into further consideration. Cytoscape 3.9.1 software (http://www.cytoscape.org/) was used to further analyze the overlapping targets. The compounds and overlapping targets were then exported into Cytoscape v3.9.1 to construct and analyze the network’s topological structures using the MCODE plug-in with the “Degree value” setting. Targets with high degree values were considered essential targets for treating OA. Kyoto Encyclopedia of Genes and Genomics (KEGG) pathway enrichment analysis and gene ontology (GO) analysis in the DAVID database were performed to investigate gene function and pathway enrichment analysis. The official gene symbols of the common targets were entered with *H. sapiens* as the selected species. Analyses yielded the top 10 GO biological processes (BP), molecular functions (MF), cellular component (CC) terms, and the top 10 KEGG pathways. We sorted the desired data by applying filters on gene *p*-value. The analysis results were visualized via R-4.1.1. The top five entries in terms of *p*-value were selected to be displayed.

### 2.8 Cell culture

RAW264.7 and SW1353 cells were purchased from Procell Life Science and Technology Co., Ltd (Wuhan, China). Passage numbers of all cells for the experiment were 5–10. The RAW264.7 cells were cultured in high-glucose Dulbecco’s Modified Eagle medium (DMEM; Gibco, MA, USA) containing 10% fetal bovine serum (FBS; Gibco), penicillin 100 U/mL (Gibco) and streptomycin 100 μg/mL (Gibco) at 37 °C in humidified conditions with 5% CO_2_. SW1353 cells were cultured in Leibovitz’s L-15 medium (Procell) containing 10% FBS, penicillin 100 U/mL, and streptomycin 100 μg/mL at 37°C in humidified conditions with no CO_2_.

### 2.9 Screening drugs for effects of anti-chondrocyte damage

The human chondrosarcoma-derived cell line SW1353 is a promising substitute for a chondrocyte experimental model. It produces sufficient proliferative activity and presents a consistent response to the phenotype of primary human chondrocytes. H_2_O_2_ stimulation induced apoptosis and necrosis, decreased cell viability, and increased eight-isoprostane F2-α expression in SW1353 cells. It is often used as a model for osteoarthritis studies (Park et al., 2018; Pang et al., 2021). For *in vitro* studies, H_2_O_2_-stimulated SW1353 cells were used as the model for chondrocyte damage, and the reversal of cell damage by drugs of interest was investigated using the CCK8 kit (Yeasen, Shanghai, China). Specifically, SW1353 cells were cultured in a complete medium containing 260 μmol/L H_2_O_2_. H_2_O_2_ stimulation and drug administration were performed simultaneously. A total of Biqi Capsule extract (10, 50, 100 μg/mL) as well as seven compounds including Strychnine, Brucine, Liquiritin, Salvianolic Acid B, Glycyrrhizic Acid, Cryptotanshinone, Tanshinone ⅡA (all concentrations are 0.2, 1, 5 μmol/L) were examined in this study. SW1353 cells were seeded with the density of 5×10^3^ cells per well in 96 well plates and incubated for 24 h before administration. The drugs were first dissolved in DMSO and then diluted with medium to the desired concentration while ensuring that the concentration of DMSO was less than 0.5‰. The DMSO was also supplemented in the Vehicle and H_2_O_2_ groups so that the DMSO concentration was the same in each group. The preparations were incubated for 12 h after administration and a CCK8 test was performed according to the manufacturer’s instructions.

### 2.10 Screening drugs for anti-inflammatory effects

For *in vitro* studies, LPS-stimulated RAW264.7 cells were used as an inflammatory model for the screening of effective anti-inflammatory drugs. LPS stimulation and drug administration were performed simultaneously. RAW264.7 cells were seeded with the density of 1×10^4^ cells per well in 96 well plates; after 24 h incubation, the culture medium was replaced with serum-free medium for another 12 h. According to the preliminary results from our pre-experiments, the cells were treated with LPS (500 ng/mL) and Biqi Capsule extract (10, 50, 100 μg/mL), Strychnine (0.1, 1, 10 μmol/L), Brucine (0.1, 1, 10 μmol/L), Liquiritin (0.1, 1, 10 μmol/L), Salvianolic Acid B (0.1, 1, 10 μmol/L), Glycyrrhizic Acid (0.1, 1, 10 μmol/L), Cryptotanshinone (0.1, 1, 10 μmol/L), Tanshinone ⅡA (0.1, 1, 10 μmol/L) for 12 h. Next, the levels of IL-6 in the supernatant were determined by ELISA according to the manufacturer’s instructions.

### 2.11 Molecular docking

The receptor is the crystal structure of RAC1, which is one of the critical targets according to the PPI network. The structure was obtained from the RCSB protein data bank, with the corresponding PDB code 1RYF. The ligands are Glycyrrhizic acid and Liquirtin, the structure files of which were obtained from PubChem.

Discovery Studio is a highly visual commercial software integrated by BIOVIA for life science research and used to perform molecular docking to investigate the interaction patterns between active compounds and critical targets. The receptor and ligands were processed by the prepare protein and prepare ligands modules, respectively, and then applied CHARMm force field on all ligands. The coordinates of the receptor active site were set to −1.23843, 73.5704, 38.9122, and a radius of 11. Parameters such as CDocker energy and CDocker interaction energy were used to evaluate the molecular docking results. The conformation with the highest CDocker interaction energy score value was selected as the most reliable binding conformation for further analysis.

### 2.12 Statistical analysis

Studies were designed to generate groups of equal size using randomization and blinded analysis. The data was presented as mean ± SEM of n measurements. N identifies the number of independent samples. Data were tested for normality using the Shapiro–Wilk test. Significant differences were assessed by either Student’s t-test or one-way ANOVA, according to the number of groups compared. When significant variations were found by one-way ANOVA, the Tukey-Kramer post-hoc test for multiple comparisons was performed only if F achieved a *p*-value <0.05. All the statistical analyses were done using GraphPad Prism 9.5. Differences were considered significant at *p* < 0.05.

## 3 Results

### 3.1 Biqi Capsule attenuates papain-induced knee OA

We assessed the efficacy of Biqi Capsule on OA using a papain-induced OA model. Firstly, no changes were observed in the rate of body weight change in Biqi Capsule treated or OA rats when compared with the Vehicle group during the 7 days (Figure 1B), which partly indicated that the Biqi Capsule treatment exerted no apparent toxic effects on OA rats. HE staining of the tissues from the knee joint of papain-treated rats showed apparent cartilage damage, mainly manifesting as localized greyish-white cartilage surface with no luster; the cartilage migration layer matrix was edematous with light red and uneven staining. Some chondrocytes showed degeneration of different sizes, clustering, and cellular disorganization; the chondrocytes in the mid and deep zones were dissolved and necrotic, with homogeneous red staining and loss of original structure, and in a few cases, there was mild hyperplasia and inflammatory cell infiltration in the synovium (Figure 1C, D). Administration of high-dose Biqi Capsule significantly ameliorated cartilage destruction and chondrocyte damage, showing similar results as celecoxib as a positive control.

Next, we measured inflammatory factors, including IL-1β, TNF-α, IL-6, and PGE2, in the synovium of rats. It was realized that Biqi Capsule significantly reduced IL-1β, IL-6, and TNF-α levels in the synovium of the OA rats (Figure 1E-G). Biqi Capsule also reduced PGE2 in the synovium, which led to hyperalgesia and allodynia (Figure 1H). Altogether, Biqi Capsule inhibited the expression and secretion of multi-cytokines, consequently inhibiting OA development.

NF-κB is a key signaling mediator of inflammation. Immunoblotting of synovial tissue showed a significant increase in the phosphorylation level of NF-κB in the OA group. These increased levels of phosphorylation were inhibited in a dose-dependent manner by Biqi Capsule (Figure 1I, J).

### 3.2 Network pharmacology analysis of Biqi Capsule

**FIGURE 2:**
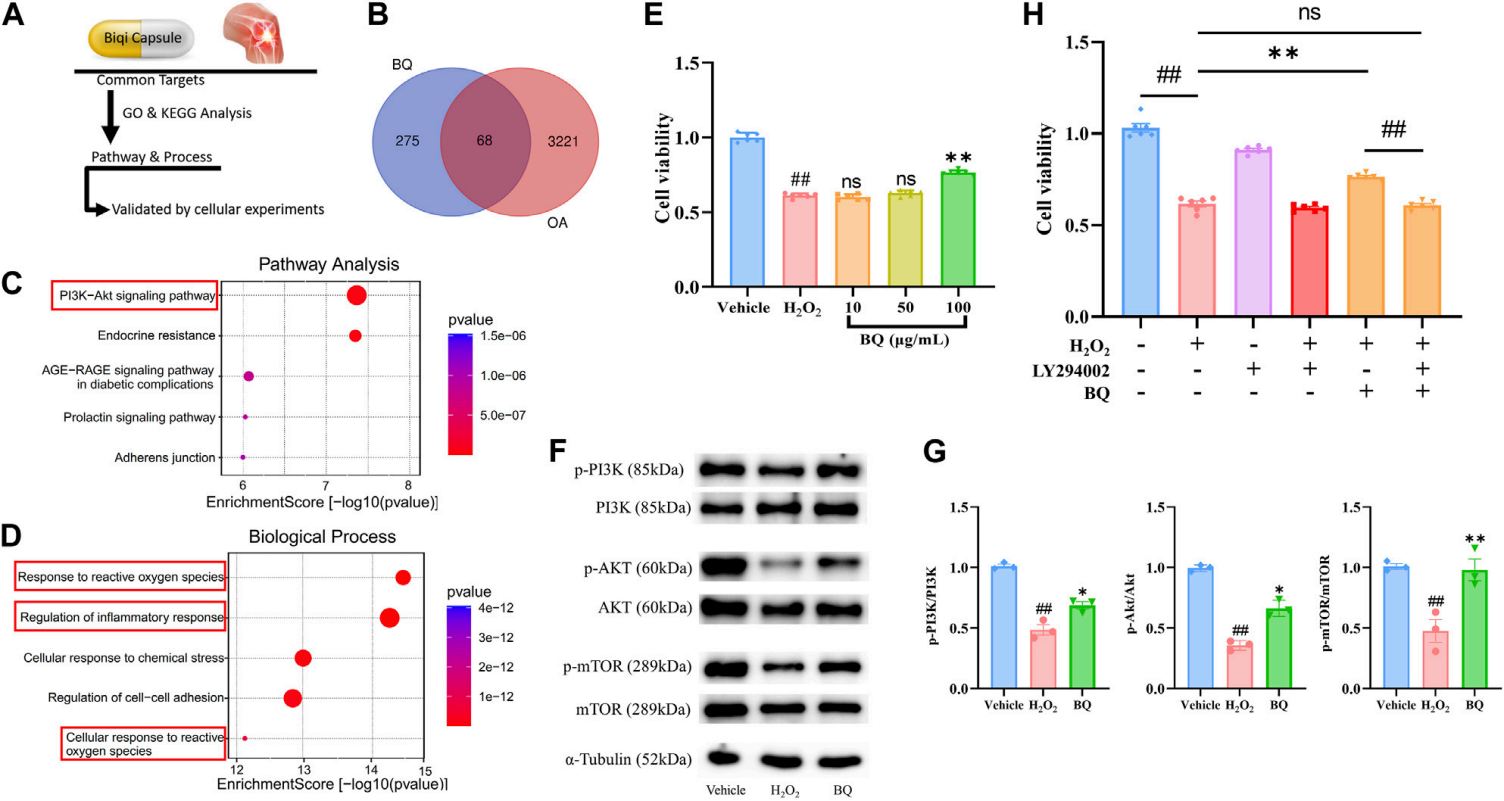
Network pharmacology analysis of Biqi Capsule for treating OA, and validated by H_2_O_2_-stimulated SW1353 cells. **(A)** Process of Network pharmacology prediction and cellular experiments validation. **(B)** Venn diagram showing overlap of OA genes and predicted compound targets. **(C)** Kyoto Encyclopedia of Genes and Genomes (KEGG) pathway enrichment analysis. **(D)** Gene Ontology (GO) analysis of biological processes. **(E)** SW1353 cells were incubated with H_2_O_2_ (260 μmol/L) and Biqi Capsule extract (BQ) for 12 h. Cell viability was determined by the CCK8 test. Data were presented as mean ± SEM. (n = 6, #*p* < 0.05, ##*p* < 0.01 compared with the Vehicle group; *p < 0.05, **p < 0.01 compared with the H_2_O_2_ group) **(F, G)** SW1353 cells were incubated with H_2_O_2_ (260 μmol/L) and Biqi Capsule extract (BQ) (100 μg/mL) for 12 h. The regulation effect of Biqi Capsule extract on phosphorylation of PI3K, AKT, and mTOR in SW1353 cells was measured by Western Blot analysis. The densitometric analyses of three independent experiments were shown. Data were presented as mean ± SEM. (n = 3, #*p* < 0.05, ##*p* < 0.01 compared with the Vehicle group; *p < 0.05, **p < 0.01 compared with the H_2_O_2_ group) **(H)** SW1353 cells were incubated with LY294002 (75 μmol/L) for 4 h and then co-incubated with H_2_O_2_ (260 μmol/L) and Biqi Capsule extract (100 μg/mL) for another 12 h. Cell viability was determined by the CCK8 test. Data were presented as mean ± SEM. (n = 6, #*p* < 0.05, ##*p* < 0.01 compared with the Vehicle group).

Network pharmacology analysis was performed to clarify the mechanisms of Biqi Capsule. Uploading seven components of Biqi Capsule to the PharmMapper server resulted in 275 candidate compound targets. By searching the OMIM, TTD, and GeneCards databases, 3221 OA-associated targets were retrieved. The 68 potential therapeutic targets of Biqi Capsule for OA were identified using Venn diagrams (Figure 2B). Furthermore, we conducted a KEGG and GO enrichment analysis on the 68 targets (Figures 2C, D). The significant and highest-scoring pathway was the PI3K/AKT pathway. Biological processes are mostly associated with inflammatory responses and oxidative stress. This suggests that the pharmacological mechanism of Biqi Capsule for the treatment of OA may be related to the inhibition of inflammatory response and oxidative stress, via PI3K/AKT signaling pathway.

### 3.3 Biqi Capsule extract reverses chondrosarcoma cell damage via PI3K/AKT/mTOR pathway

Network Pharmacology showed that the PI3K/AKT pathway is a critical pathway for Biqi Capsule in the treatment of OA. Activated PI3K/AKT/mTOR signaling has been found in many studies to promote chondrocyte proliferation and reduce cartilage damage (Sun et al., 2020). To confirm the anti-chondrocyte damage effect of Biqi Capsule, we examined the effect of Biqi Capsule extract on chondrosarcoma cell line SW1353 cell damage induced by H_2_O_2_ (Figure 2E). After H_2_O_2_ stimulation, the cell viability was significantly reduced, in association with the reduction of phosphorylation of PI3K, AKT, and mTOR. Biqi Capsule extract (100 μg/mL) increased the viability in H_2_O_2_-stimulated SW1353 cells. Mechanically, Biqi Capsule extract (100 μg/mL) upregulated the phosphorylation of PI3K, AKT, and mTOR in H_2_O_2_-stimulated SW1353 cells (Figure 2E∼G). According to one study, PI3K inhibitor LY294002 could strongly inhibit the activation of PI3K in SW1353 cells both *in vivo* and *in vitro* (Huang et al., 2017; Xu et al., 2019; Zhu et al., 2019; Xu et al., 2020). In line with our current study, it was revealed that Biqi Capsule extract could not reverse the H_2_O_2_-stimulated damage in SW1353 cells that were treated with LY294002 (Figure 2H). Given these findings, we assumed that Biqi Capsule could ameliorate OA by stimulating, as well as enhancing the activity of the PI3K/AKT/mTOR pathway in knee joint cartilage.

### 3.4 Screening for bioactive components on H_2_O_2_-Stimulated SW1353 cells

**FIGURE 3:**
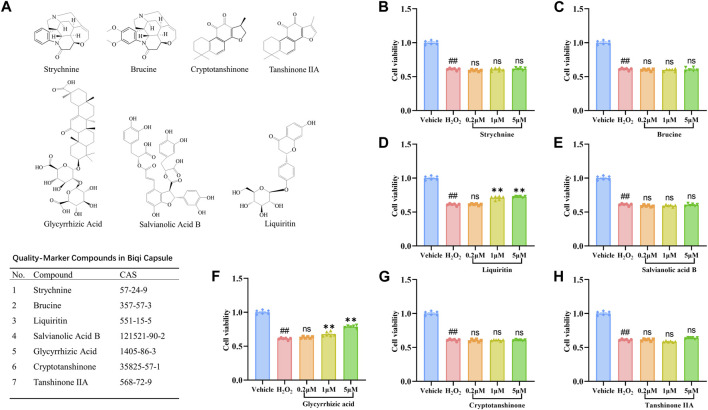
Effect of seven compounds of Biqi Capsule on the viability of H_2_O_2_-stimulated SW1353 cells. **(A)** The chemical structures of seven Quality-markers of Biqi Capsule. **(B–H)** SW1353 cells were incubated with H_2_O_2_ (260 μmol/L) and Strychnine/Brucine/Liquiritin/Salvianolic Acid B/Glycyrrhizic Acid/Cryptotanshinone/Tanshinone ⅡA for 12h, respectively. Cell viability was determined by the CCK8 test. Data were presented as mean ± SEM. (n = 6, #*p* < 0.05, ##*p* < 0.01 compared with the Vehicle group; *p < 0.05, **p < 0.01 compared with the H_2_O_2_ group).

To explore the active components of Biqi Capsule against chondrosarcoma cell damage, we screened for the effects of the seven components of Biqi Capsule, including Strychnine, Brucine, Liquiritin, Salvianolic Acid B, Glycyrrhizic Acid, Cryptotanshinone, Tanshinone ⅡA on the viability of H_2_O_2_-stimulated SW1353 cells. Among these components, Glycyrrhizic Acid and Liquiritin were found to increase cell viability. Moreover, the effects of Glycyrrhizic Acid and Liquiritin were also found to be dose-dependent (Figure 3).

### 3.5 Regulation of PI3K/AKT/mTOR pathway by Glycyrrhizic Acid and Liquiritin

**FIGURE 4:**
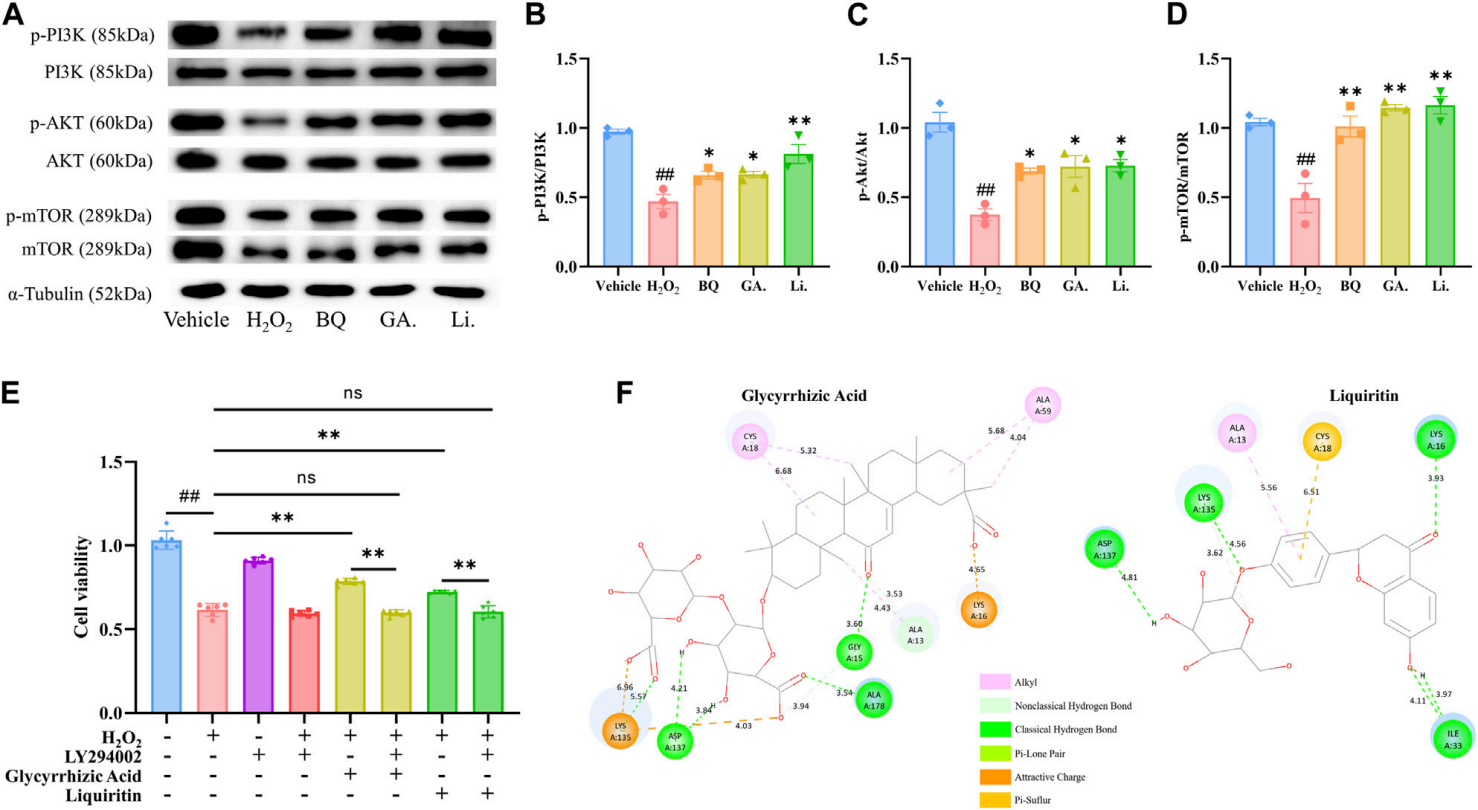
Effect of Glycyrrhizic Acid and Liquiritin on H_2_O_2_-stimulated SW1353 cells. **(A–D)** SW1353 cells were incubated with H_2_O_2_ (260 μmol/L) and Glycyrrhizic Acid (GA) (5 μmol/L)/Liquiritin (Li.) (5 μmol/L) for 12 h. The regulation effect of Biqi Capsule extract (BQ), Glycyrrhizic Acid, and Liquiritin on phosphorylation of PI3K, AKT, and mTOR in SW1353 cells was measured by western blot analysis. The densitometric analyses of three independent experiments were shown. Data were presented as mean ± SEM. (n = 3, #*p* < 0.05, ##*p* < 0.01 compared with the Vehicle group; *p < 0.05, **p < 0.01 compared with the H_2_O_2_ group) **(E)** SW1353 cells were incubated with LY294002 (75 μmol/L) for 4 h and then co-incubated with H_2_O_2_ (260 μmol/L) and Glycyrrhizic Acid (5 μmol/L)/Liquiritin (5 μmol/L) for another 12 h. Cell viability was determined by the CCK8 test. Data were presented as mean ± SEM. (n = 6, #*p* < 0.05, ##*p* < 0.01 compared with the Vehicle group) **(F)** Docking structure of active compounds with RAC1.

The treatment with Glycyrrhizic Acid (5 μmol/L) and Liquiritin (5 μmol/L) resulted in a significant increase in cell viability (Figure 3D–F), so we investigated whether Glycyrrhizic Acid (5 μmol/L) and Liquiritin (5 μmol/L) could regulate the PI3K/AKT/mTOR pathway. Glycyrrhizic Acid and Liquiritin upregulated the phosphorylation of PI3K, AKT, and mTOR in H_2_O_2_-stimulated SW1353 cells (Figure 4A–D). Moreover, PI3K inhibitor blocks the protective effect of Glycyrrhizic Acid and Liquiritin on H_2_O_2_-induced SW1353 cells (Figure 4E).

### 3.6 Interaction of bioactive compounds with RAC1

In the PPI network, we obtained one key core target, RAC1, which is an upstream target of the PI3K/AKT/mTOR pathway. Glycyrrhizic Acid and Liquiritin treatment modulated the PI3K/AKT/mTOR pathway. Thus, we speculated that Glycyrrhizic Acid and Liquiritin actively target RAC1 to reduce chondrocyte damage via the RAC1/PI3K/AKT/mTOR pathway. To confirm this, we performed molecular docking studies to gain insight into the binding modes of Glycyrrhizic Acid and Liquiritin upon binding to vital regulatory sites on RAC1, respectively. A negative value of CDocker Energy indicates that the ligand-receptor has decent binding activity. We found that the CDocker Energy of Glycyrrhizic Acid and Liquiritin with RAC1 were −91.3 and −53.9 respectively, which are all much less than zero, indicating that the docking structures of these compounds with RAC1 are considerably stable. We selected the docking structure with the lowest CDocker interaction energy after docking with RAC1 for further analysis. Figure 4F shows the chemical bonds and distances between the different atomic groups of each compound interacting separately with the different amino acids of the RAC1 protein. Glycyrrhizic Acid forms six hydrogen bonds with A13, A15, A137 and A178, Liquiritin forms six hydrogen bonds with A16, A33, A135 and A137. This finding indicates that the Glycyrrhizic Acid and Liquiritin may induce a conformational change in RAC1 that promotes binding to other downstream effectors.

### 3.7 Screening for active components on LPS-stimulated RAW264.7 cells

**FIGURE 5:**
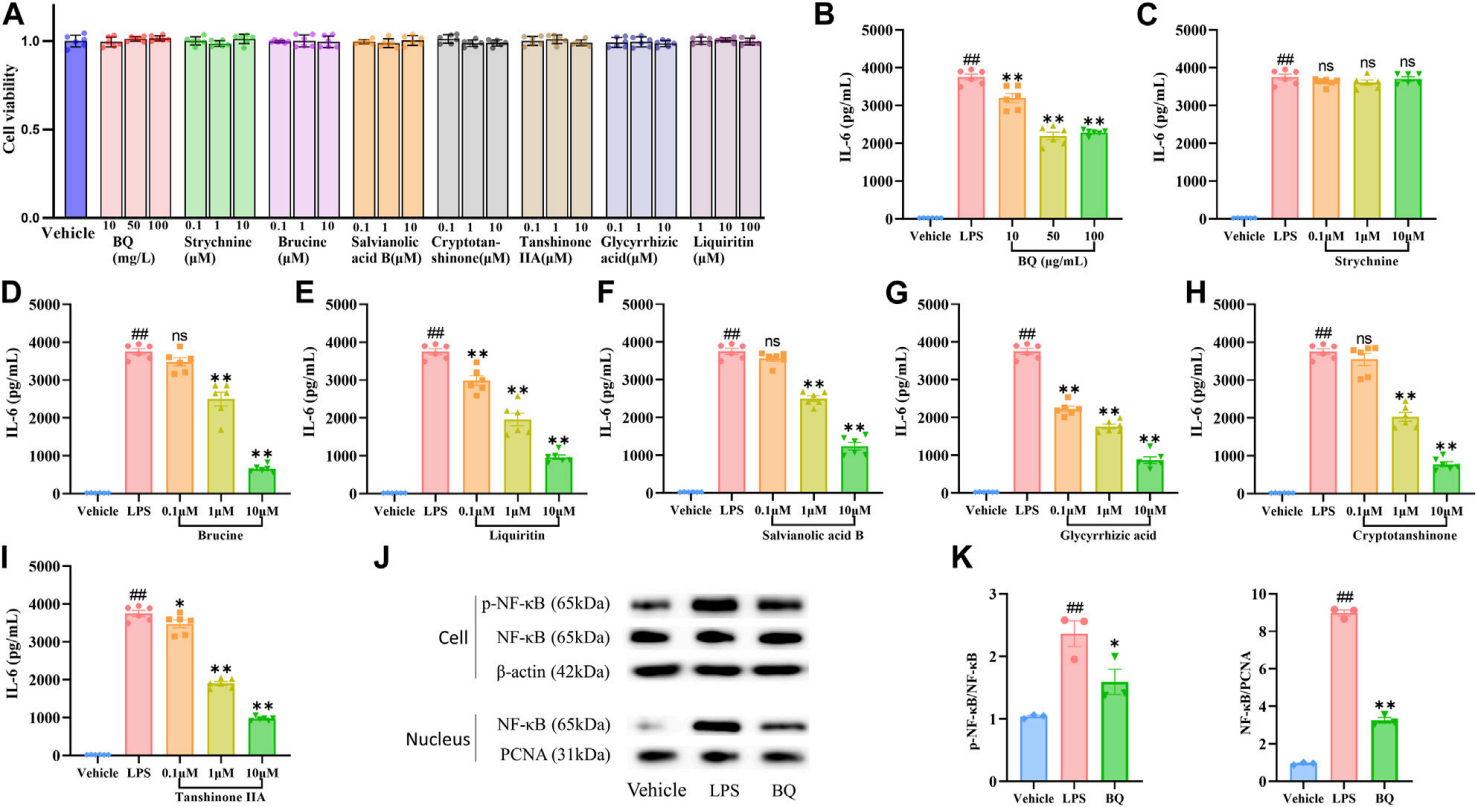
Effect of Biqi Capsule extract and seven compounds on the LPS-stimulated RAW264.7 cells. **(A)** RAW264.7 cells were incubated with drugs for 12h, respectively. Drug cytotoxicity was determined by the CCK-8 test. Data were presented as mean ± SEM. (n = 6) **(B–I)** RAW264.7 cells were incubated with LPS (500 ng/mL) and Biqi Capsule extract (BQ)/Strychnine/Brucine/Liquiritin/Salvianolic Acid B/Glycyrrhizic Acid/Cryptotanshinone/Tanshinone ⅡA for 12 h. IL-6 in the supernatant was examined by ELISA. Data were presented as mean ± SEM. (n = 6, #*p* < 0.05, ##*p* < 0.01 compared with the Vehicle group; *p < 0.05, **p < 0.01 compared with the LPS group) **(J, K)** RAW264.7 cells were incubated with LPS (500 ng/mL) and Biqi Capsule extract (BQ) (50 μg/mL) for 12 h. Nuclear and cytoplasm protein extractions of RAW264.7 cells were conducted according to the manual of the Nuclear Protein Extraction Kit (Solarbio, Beijing, China). The regulation effect of Biqi Capsule extract on phosphorylation and nuclear translocation of NF-κB (p65) in RAW264.7 cells was measured by western blot analysis. The purity of the nuclear protein was validated via western blotting with anti-β-actin. Data were presented as mean ± SEM. (n = 3, #*p* < 0.05, ##*p* < 0.01 compared with the Vehicle group; *p < 0.05, **p < 0.01 compared with the LPS group).

*In vivo* experiments and network pharmacology analysis showed the anti-inflammatory effect of Biqi Capsule. Thus, we examined the effects of Biqi Capsule extract and the seven compounds on the expression of IL-6 in LPS-stimulated RAW264.7 cells. The CCK8 test showed that none of the experimental concentrations of all drugs used were toxic to RAW264.7 cells (Figure 5A). After LPS stimulation, the IL-6 concentrations were significantly increased. Treatment with Biqi Capsule extract, Brucine, Liquiritin, Salvianolic acid B, Glycyrrhizic Acid, Cryptotanshinone, and Tanshinone ⅡA decreased IL-6 production (Figure 5B, D–I). In addition, the effect was found to be dose-dependent. Among these compounds, Glycyrrhizic Acid showed the most potent anti-inflammatory activity with EC_50_: 2.25 μmol/L.

### 3.8 Biqi Capsule attenuates NF-κB activation *in vitro*

NF-κB signaling plays a central role in pro-inflammatory stress-related responses, which also mediates the expression of IL-6. To determine whether the anti-IL-6 effect of Biqi Capsule extract was related to NF-κB, we first detected the activation of NF-κB in RAW264.7 cells. Nuclear translocation and phosphorylation of NF-κB lead to the transcription of genes encoding proinflammatory mediators. Immunoblotting showed that the nuclear translocation and phosphorylation (Figure 5J–K) of NF-κB (p65) were all significantly increased after LPS stimulation. Notably, nuclear translocation and phosphorylation of NF-κB were inhibited by Biqi Capsule extract. In addition, the Biqi Capsule also inhibited the phosphorylation of NF-κB and the expression of IL-6 in OA rats. Taking these findings into consideration, we suggest that the therapeutic effect of Biqi Capsule on the inflammatory response in OA is achieved via the NF-κB signaling pathway, and the bioactive components responsible for this observable effect are Brucine, Liquiritin, Salvianolic Acid B, Glycyrrhizic Acid, Cryptotanshinone, and Tanshinone ⅡA.

## 4 Discussion and conclusion

This study demonstrated the pharmacological mechanism and bioactive components of Biqi Capsule in the treatment of OA. The novel findings are as follows: 1. Biqi Capsule has chondroprotective and anti-synovitis effect in papain-induced OA rats. 2. Biqi Capsule exerts a chondroprotective effect by activating the PI3K/AKT/mTOR pathway in chondrocytes, and the relevant bioactive components are Glycyrrhizic Acid and Liquiritin. 3. Biqi Capsule exerts an anti-inflammatory effect by inhibiting the NF-κB/IL-6 pathway, and the relevant bioactive components are Brucine, Liquiritin, Salvianolic Acid B, Glycyrrhizic Acid, Cryptotanshinone, Tanshinone ⅡA. 4. Glycyrrhizic Acid and Liquiritin can be considered novel lead compounds for the treatment of osteoarthritis.

OA is a chronic progressive disease characterized by degenerative changes in the knee joint cartilage. Conventionally, the commonly used classes of drugs for the treatment of OA include NSAIDs, locally administered corticosteroids, analgesics, etc. The long-term use of these drugs gradually leads to obvious adverse effects that some patients cannot tolerate (Brophy and Fillingham, 2022). Biqi Capsules is used to treat many types of arthritis in Traditional Chinese medicine with proven efficacy and safety (Chen et al., 2018; Wang et al., 2020). A study investigated the effects of Biqi Capsule on CIA-induced rheumatoid arthritis in rats. The study revealed that Biqi Capsule alleviated local joint and systemic inflammation, synovial hyperplasia, and cartilage destruction caused by CIA (Wang et al., 2018). In this current study, we demonstrated that Biqi Capsule could resist the progression of papain-induced OA. Biqi Capsule improved joint function in rats, inhibited the expression of inflammatory factors in the synovium, and protected cartilaginous tissues from destruction.

PI3K/AKT signaling negatively modulates chondrocyte apoptosis under multiple pathological conditions, and the activated signaling can prevent OA by reducing chondrocyte apoptosis. Clinical studies have revealed that the PI3K/AKT pathway is downregulated in human cartilage tissues with OA when compared with normal cartilage (Rosa et al., 2011; Wang et al., 2020). Similarly, OA-like chondrocytes exposed to inflammation or oxidative stress showed downregulation of PI3K/AKT activity. It has been suggested that E2-mediated PI3K/AKT activation significantly promotes the proliferation and viability of chondrocytes in OA rat model and ATDC5 chondrocytes (Huang et al., 2011; Fan et al., 2018). In this study, we first analyzed the possible mechanisms of Biqi Capsule for OA by employing network pharmacology. This revealed that PI3K/AKT might be the main target pathway of Biqi Capsule against cartilage damage. Consistent with the analysis, we validated that Biqi Capsule extract activates the PI3K/AKT and its downstream protein mTOR in H_2_O_2_-stimulated chondrocytes. Further, we performed PI3K/AKT/mTOR pathway inhibition experiments to highlight the chondroprotective mechanism of Biqi Capsule extract, which is mainly focused on the PI3K/AKT/mTOR axis. Furthermore, we screened seven compounds of Biqi Capsule. Firstly, we found that Glycyrrhizic Acid and Liquiritin activated the PI3K/AKT/mTOR pathway in H_2_O_2_-stimulated SW1353 cells and also inhibited cell death. Some studies have implied that both overactivation and inhibition of RAC1 in cartilage tissues lead to pathological effects, and regulation of RAC1 signaling is crucial for maintaining homeostasis in cartilaginous tissues (Woods et al., 2009; Suzuki et al., 2017). Meanwhile, the network pharmacology analysis (Supplementary Material S2) revealed that RAC1 is the key target protein responsible for the therapeutic effects observed in the treatment of osteoarthritis with Biqi Capsule; and is also intricately associated with the PI3K/AKT/mTOR pathway. Previous studies have suggested that PI3K is a direct target of activated RAC1: RAC1 interacts with the BCR homology domain of the PI3K regulatory subunit p8593, and this interaction is significantly enhanced when RAC1 is bound to GTP (Bokoch et al., 1996; Campa et al., 2015). Consequently, we performed molecular docking experiments on GTP-RAC1 binding site and two compounds that are known for their chondroprotective effects, Glycyrrhizic Acid and Liquiritin, to investigate their potential interactions with this binding site. The docking results demonstrated strong interactions of Glycyrrhizic Acid and Liquiritin with the site (−91.3 kcal/mol and −53.9 kcal/mol, respectively), with docking structures similar to that of GTP-RAC1 structure. Therefore, we suggest that Glycyrrhizic Acid and Liquiritin in Biqi Capsule exert their chondroprotective effects by activating RAC1 in chondrocytes, consequently activating the PI3K/AKT/mTOR pathway in these cells. Glycyrrhizic Acid and Liquiritin are both natural constituents derived from licorice. Both are known for their low toxicity and wide range of pharmacological activities. Glycyrrhizic acid, in particular exhibits favorable pharmacokinetic characteristics and has been extensively studied, leading to its development as a hepatoprotective drug in Japan and China for cases of chronic hepatitis. However, the development of Liquiritin as a drug has been faced with challenges due to its low bioavailability and limited absorption. Despite this, Liquiritin shows promise in preventing premature delivery caused by progesterone deficiency, as it acts as a strong selective inhibitor of AKR1C1, a key enzyme in progesterone metabolism (Ming and Yin, 2013; Qin et al., 2022). As far as we know, this is the first time that Glycyrrhizic Acid and Liquiritin are being suggested to exert chondroprotective effects via PI3K/AKT/mTOR pathway activation.

On the other hand, the inflammatory response that occurs in joints affected by osteoarthritis, synoviocytes are stimulated by cytokines and matrix fragments derived from cartilage degeneration and cellular stress products which activate the NF-κB signaling pathway via the TL-R and chemokine surface receptors (Midwood et al., 2009; Liu et al., 2012; Molnar et al., 2021). Afterward, the activated NF-κB mediates the synthesis of cytokines like IL-1β, IL-6, TNF-α, PGE2, MMPs1-13, ADAMTS4, ADAMTS5, chemokines like IL-8, CCL5, and angiogenic factors like VEGF and bFGF, which cause further cartilage degradation and inflammation of the synovium. Notably, NF-κB cooperates with activated AP-1 to mediate IL-1β-induced MMP1 and MMP3 expression as well as CTGF-triggered IL-6 production. Synovitis is then accompanied by a series of histological abnormalities that occur in the synovium, such as infiltration of macrophages and lymphocytes, joint swelling, and stiffness. The definitive role of NF-κB in OA onset and progression provides evidence that interventions targeted at this signaling pathway might have beneficial therapeutic effects (Rigoglou and Papavassiliou, 2013; Burke et al., 2019; Molnar et al., 2021). The non-steroidal anti-inflammatory drugs (NSAIDs) and glucocorticoids are some of the pharmacologically active compounds that hinder the activation of NF-κB cascades. Inhibition of IL-6 activity reduces the severity of experimental OA (Latourte et al., 2017; Ansari et al., 2019). Regarding the anti-inflammatory effects of Biqi Capsule, a previous study indicated that Biqi Capsule could resist the inflammatory response of LPS-stimulated RAW264.7 cells via the NO-iNOS pathway and COX-2 pathway (Wang et al., 2011). Our study complements these previous findings by elucidating the regulatory role of Biqi Capsule on the NF-κB/IL-6 pathway in synovial tissue and macrophage. Per previous studies, the bioactive components of Biqi Capsule, Strychnine, Brucine, Cryptotanshinone, Glycyrrhizic Acid, and Liquiritin have been found to inhibit NO secretion on LPS-stimulated macrophages, while it was only cryptotanshinone that inhibited IL-6 expression (Qishuai et al., 2016). Our study supplemented the previous results by identifying the possible anti-inflammatory effect of Brucine, Liquiritin, Salvianolic Acid B, Glycyrrhizic Acid, Cryptotanshinone, Tanshinone ⅡA, which have already been proven to exert similar anti-inflammatory effects in a wide range of diseases (Yin et al., 2003; Gao et al., 2019; Zhai et al., 2019; Wu et al., 2020; Xiao et al., 2020; *Li* et al., 2021; Wang et al., 2022).

**FIGURE 6:**
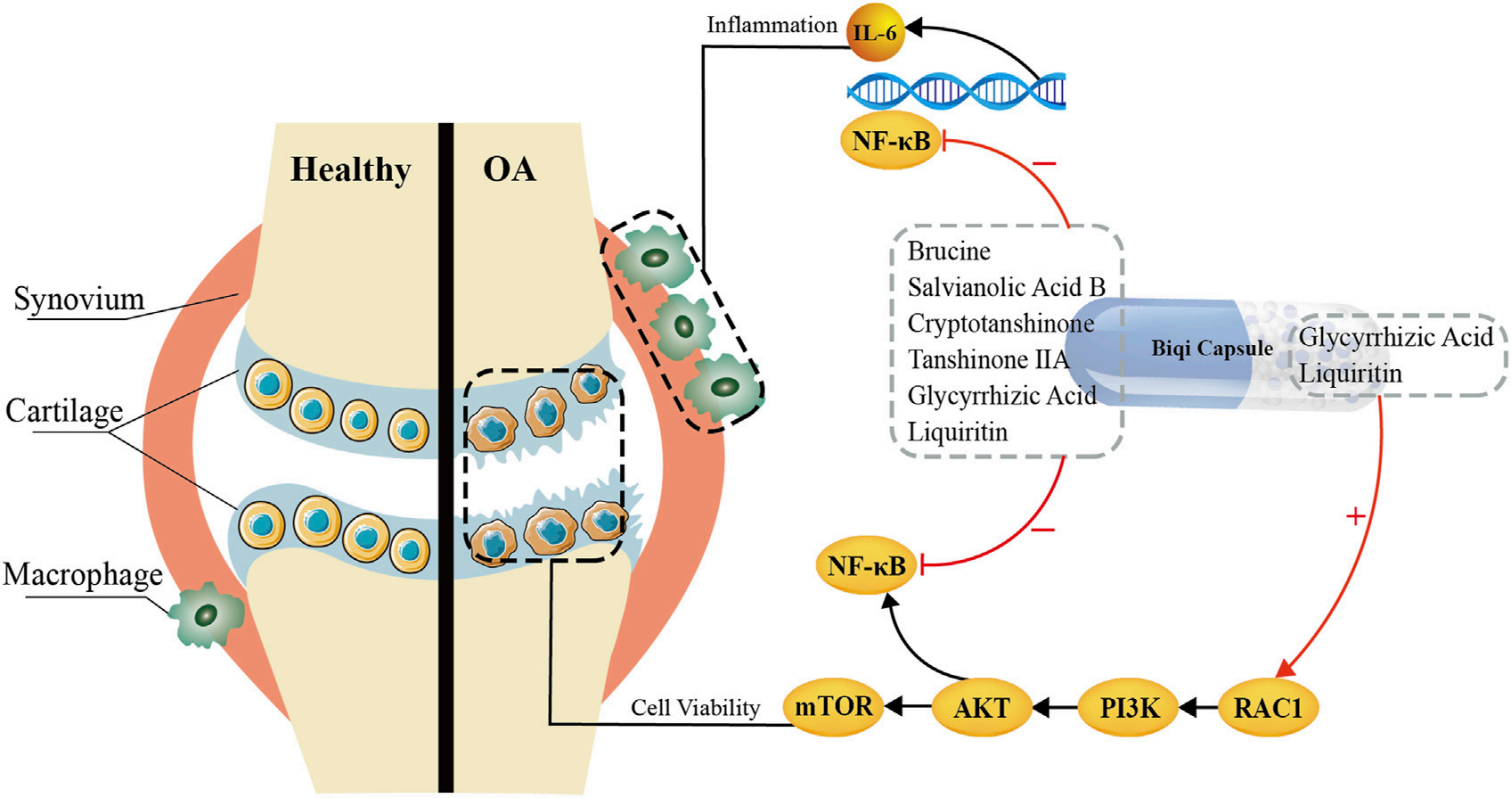
Bioactive Components and Potential Mechanisms of Biqi Capsule in the Treatment of Osteoarthritis.

In addition, the PI3K/AKT signaling pathway, as the upstream pathway of NF-κB, is associated with inflammation in osteoarthritis to some extent. Previous studies have shown that some medicines (like the Huoxuezhitong capsule) can treat the inflammation by inhibiting the PI3K/AKT/NF-κB signaling pathway (Chen et al., 2013; Wang et al., 2016; Ju et al., 2020). This seems to be contrary to our current findings which suggest that Glycyrrhizic Acid and Liquiritin in Biqi Capsule treat osteoarthritis by activating PI3K/AKT. Both mTOR and NF-κB are downstream proteins of the PI3K/AKT signaling pathway. It appears that Glycyrrhizic Acid and Liquiritin, while activating mTOR via the PI3K/AKT pathway, would incidentally lead to the activation of NF-κB, resulting in an increased inflammatory response. However, our *in vivo* experiments showed that the chondroprotective effect of Biqi capsules did not exacerbate or even inhibit the inflammatory response. This may be due to the ability of active ingredients like Brucine in Biqi Capsule to inhibit the phosphorylation and nuclear translocation of NF-κB. This also demonstrates the characteristic “multi-component/multi-target/multi-pathway” of Traditional Chinese Medicines in the treatment of diseases. In conclusion, we have found that Glycyrrhizic Acid and Liquiritin in Biqi Capsule can activate the PI3K/AKT pathway, leading to the activation of the mTOR and the consequent treatment of cartilage damage. Additionally, components including Brucine can inhibit NF-κB activation, whether it is triggered by the activation of the PI3K/AKT pathway or by osteoarthritis itself (Figure 6).

There are several caveats in this study. Firstly, the pharmacological mechanism of the drugs *in vivo*, and whether components other than Glycyrrhizic Acid and Liquiritin have a chondroprotective effect, still need to be further explored considering the genetic differences between cell lines and natural cells or living organisms. Furthermore, although we discovered that Biqi Capsule extract, Glycyrrhizic Acid, and Liquiritin can activate the PI3K/AKT/mTOR pathway to resist H_2_O_2_-induced cell death (e.g., apoptosis, autophagy, etc.), yet it is unclear which mode of cell death is regulated by these drugs. Thus, further identification of signature proteins for a particular mode of cell death is warranted. What’s more, no anti-inflammatory or chondroprotective effect of Strychnine was observed. In light of this, further investigation regarding Strychnine as an anti-inflammatory or chondroprotective agent is needed.

Overall, based on the anti-inflammatory and anti-cartilage damage effects of Biqi Capsule in OA rats, we suggest that Glycyrrhizic Acid and Liquiritin can exert chondroprotective effects by activating the PI3K/AKT/mTOR pathway; Brucine, Liquiritin, Salvianolic Acid B, Glycyrrhizic Acid, Cryptotanshinone, Tanshinone ⅡA can modulate the NF-κB/IL-6 pathway in macrophages and synovial tissues to exert anti-inflammatory effects. This may provide empirical evidence and a theoretical foundation for the clinical application of Biqi Capsule as well as the development of Glycyrrhizic Acid and Liquiritin as anti-OA lead compounds. Biqi Capsule is a promising therapeutic option for the treatment of OA.

## Data Availability

The original contributions presented in the study are included in the article/Supplementary Material, further inquiries can be directed to the corresponding authors.
