# Supplementary material for: Bioactive components and potential mechanisms of Biqi Capsule in the treatment of osteoarthritis: based on chondroprotective and anti-inflammatory activity
Source: Front Pharmacol. 2024 Apr 17;15:1347970. doi: 10.3389/fphar.2024.1347970 (PMC11061359; doi:10.3389/fphar.2024.1347970)

The whole images of the original western blots (For Figure 1E).

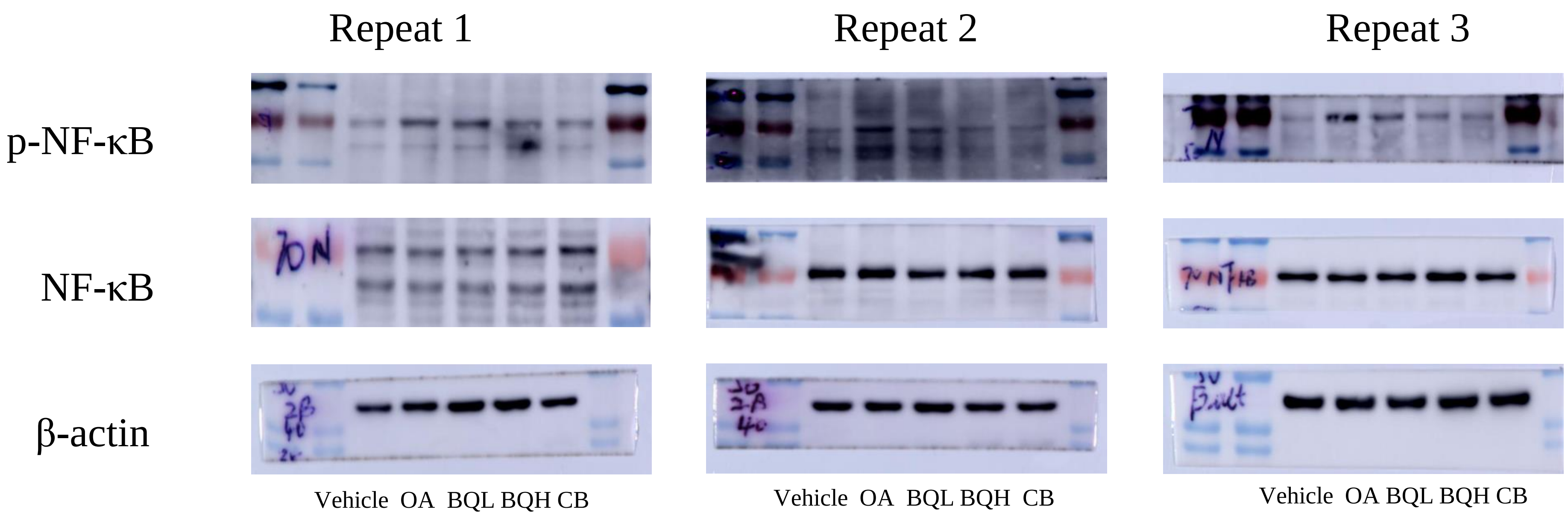

The whole images of the original western blots (For Figure 2F and Figure 4A).

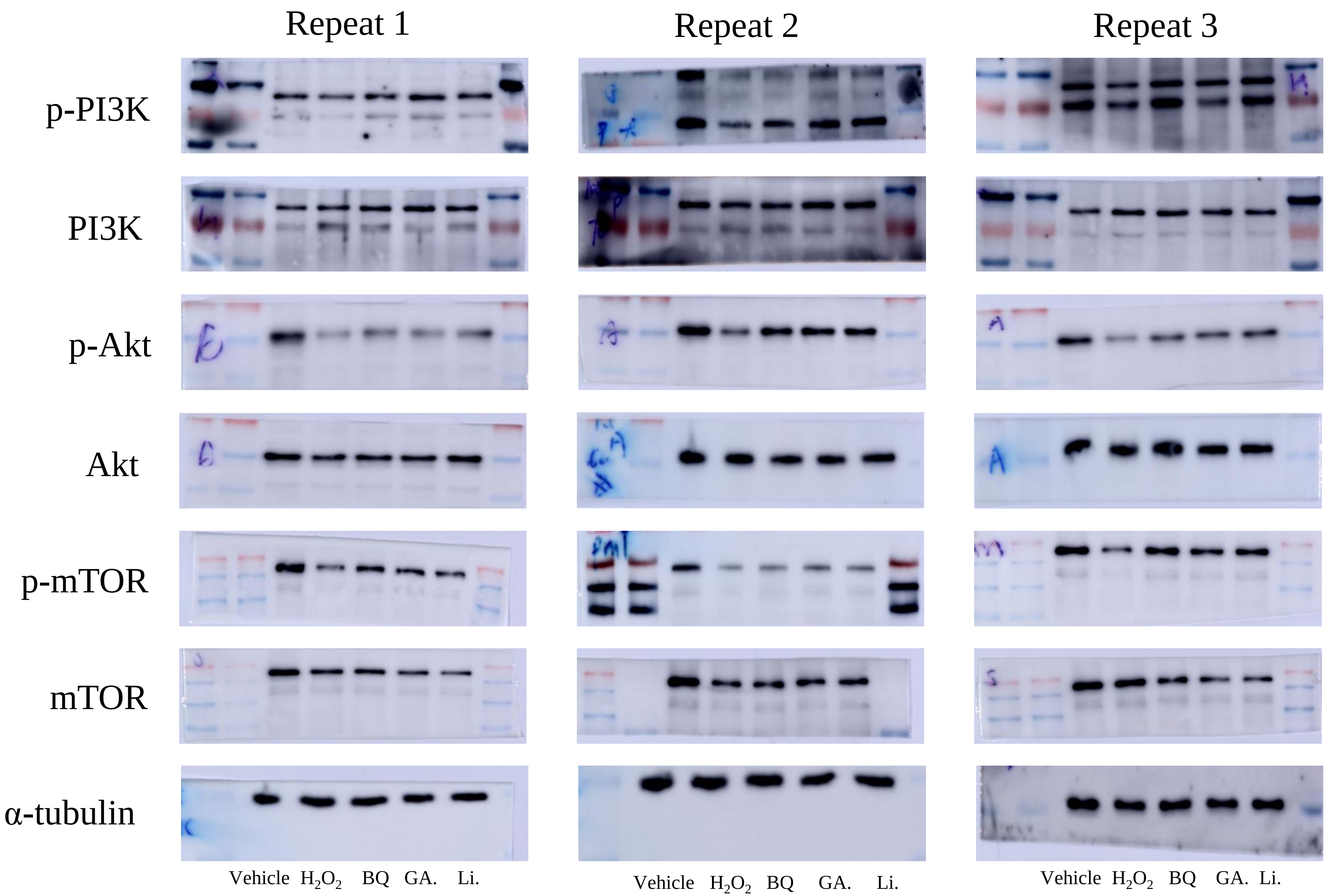

The whole images of the original western blots (For Figure 5J).

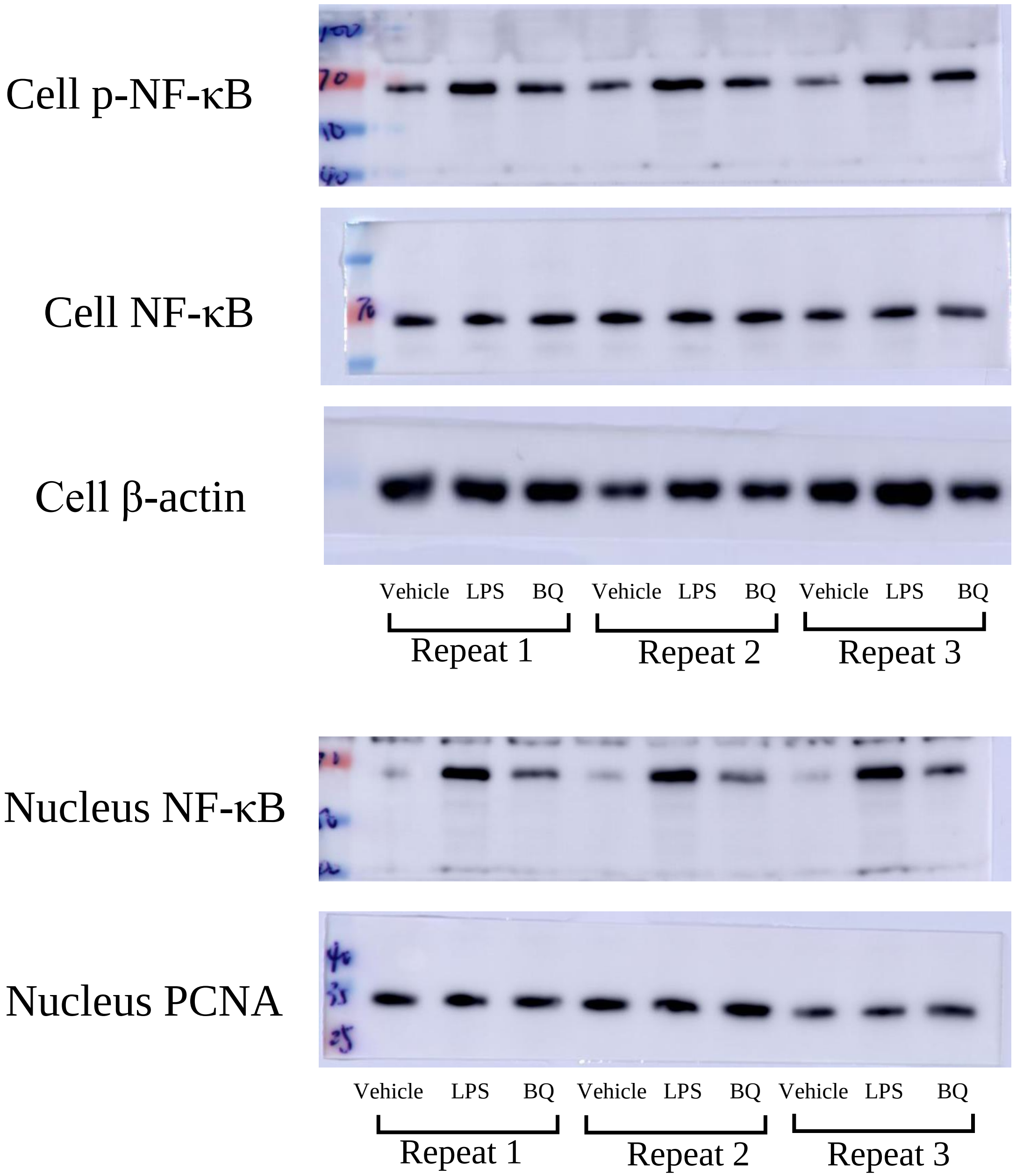

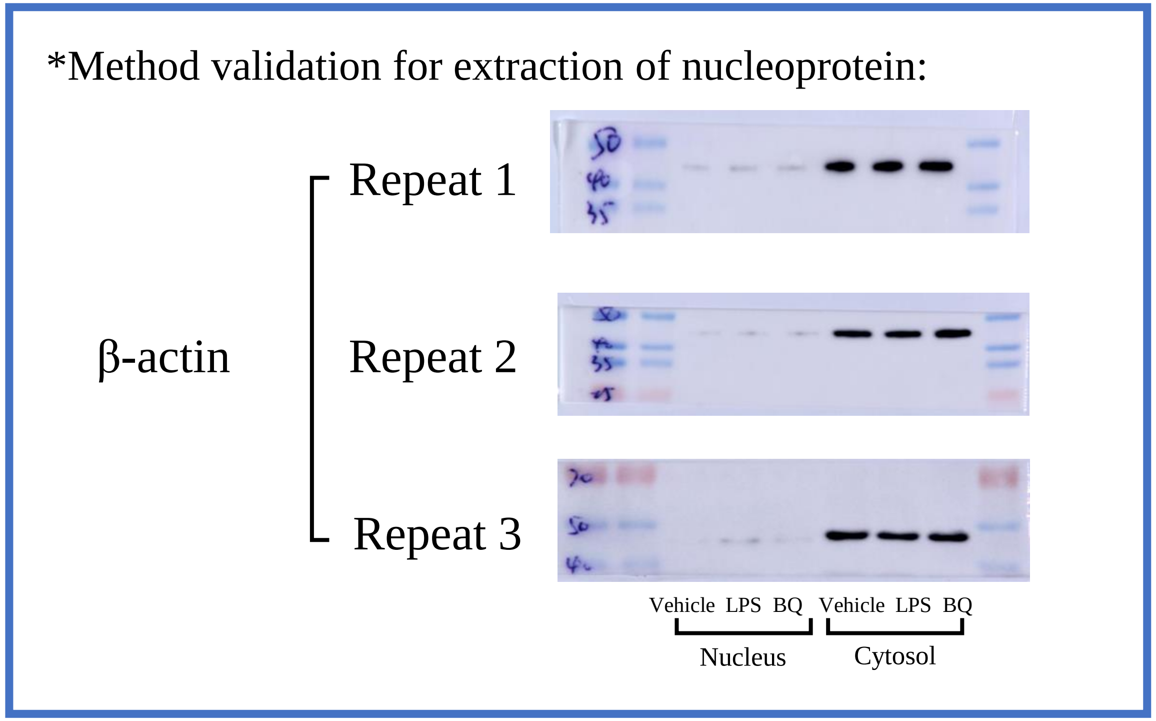

Supplement: Supplementary file 1 [file DataSheet2.PDF]
