# Supplementary material for: Bioactive components and potential mechanisms of Biqi Capsule in the treatment of osteoarthritis: based on chondroprotective and anti-inflammatory activity
Source: Front Pharmacol. 2024 Apr 17;15:1347970. doi: 10.3389/fphar.2024.1347970 (PMC11061359; doi:10.3389/fphar.2024.1347970)

Supplementary material 1:

HE-stained sections of knee joints from rats in different groups (For Figure 1C).

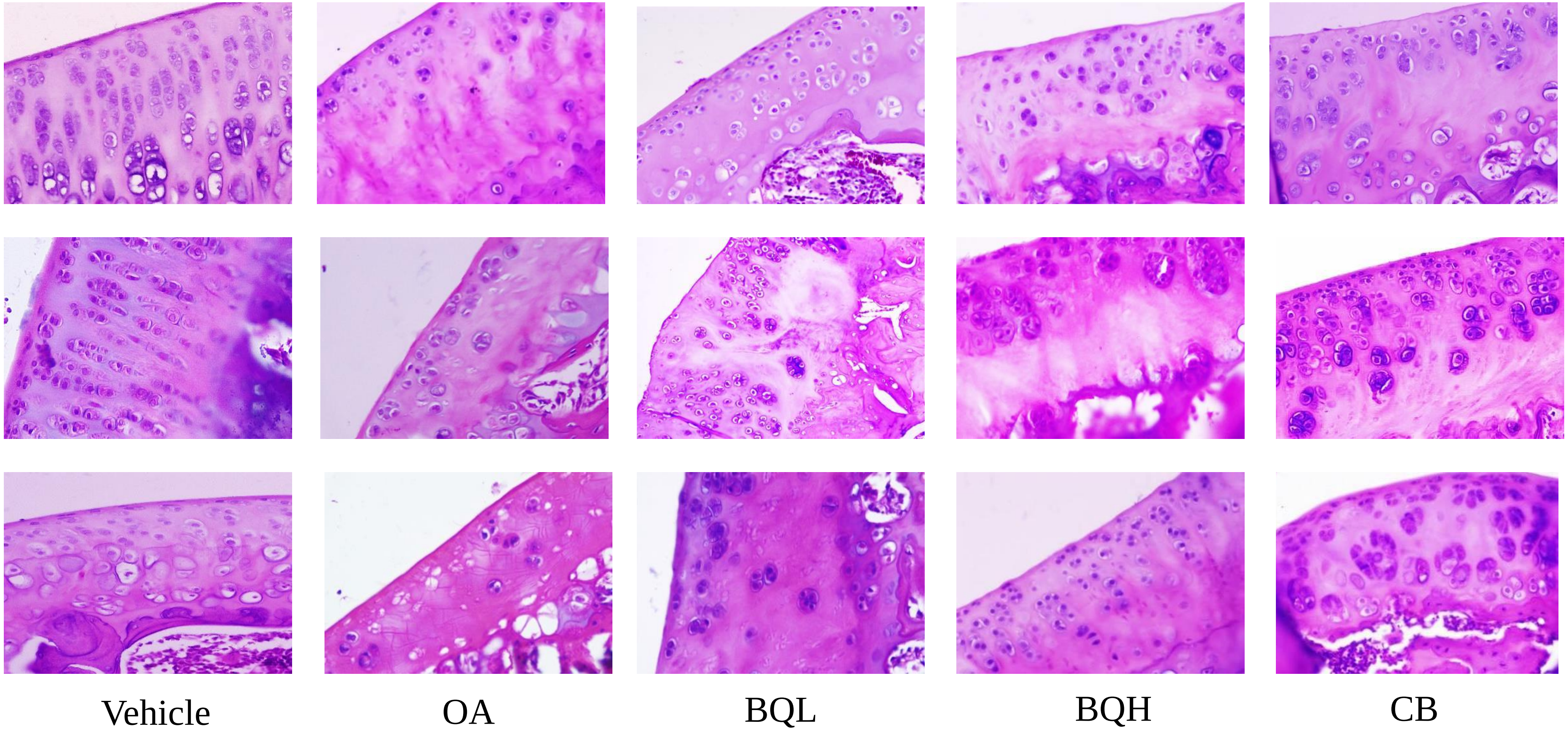

Supplementary material 2:

The network relationship among compounds and predicted targets. Green nodes represent compounds of Biqi Capsule, and black nodes represent OA targets.

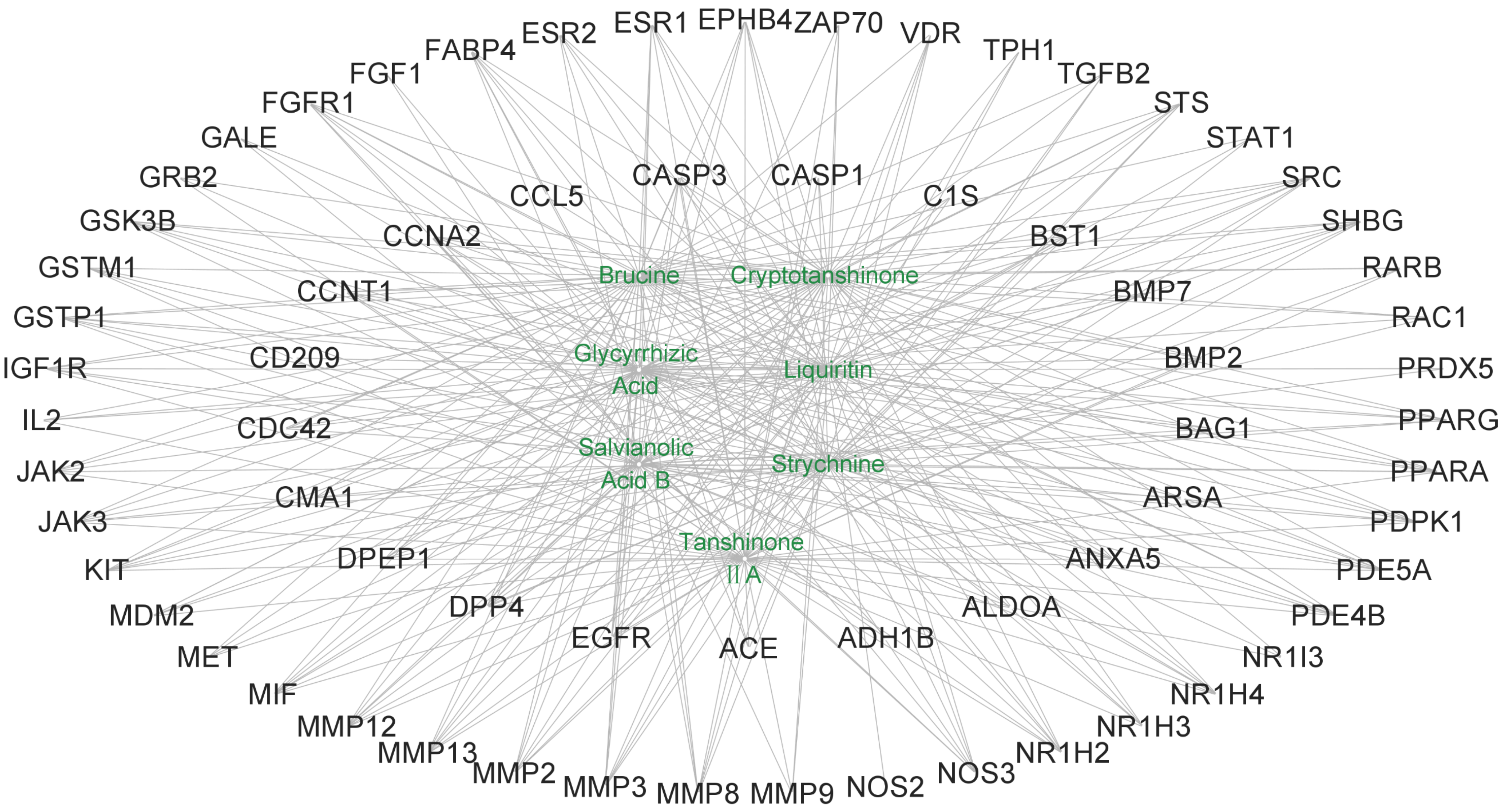

Supplementary material 3:

The whole images of the original western blots (For Figure 1E).

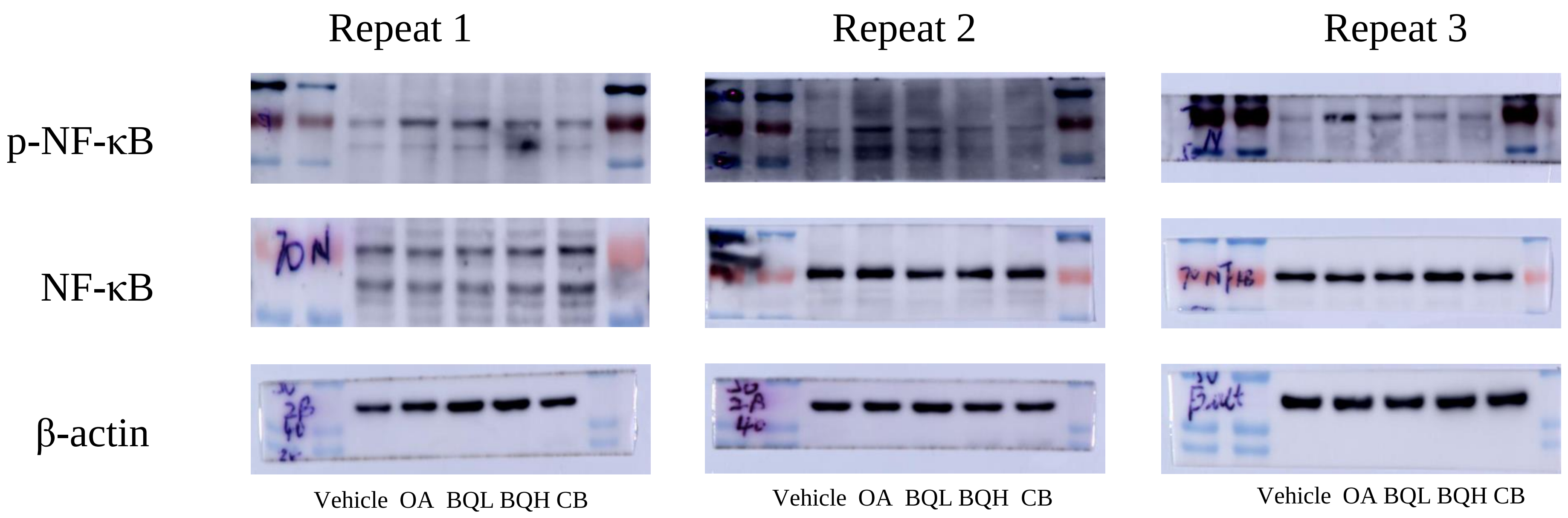

Supplementary material 4:

The whole images of the original western blots (For Figure 2F and Figure 4A).

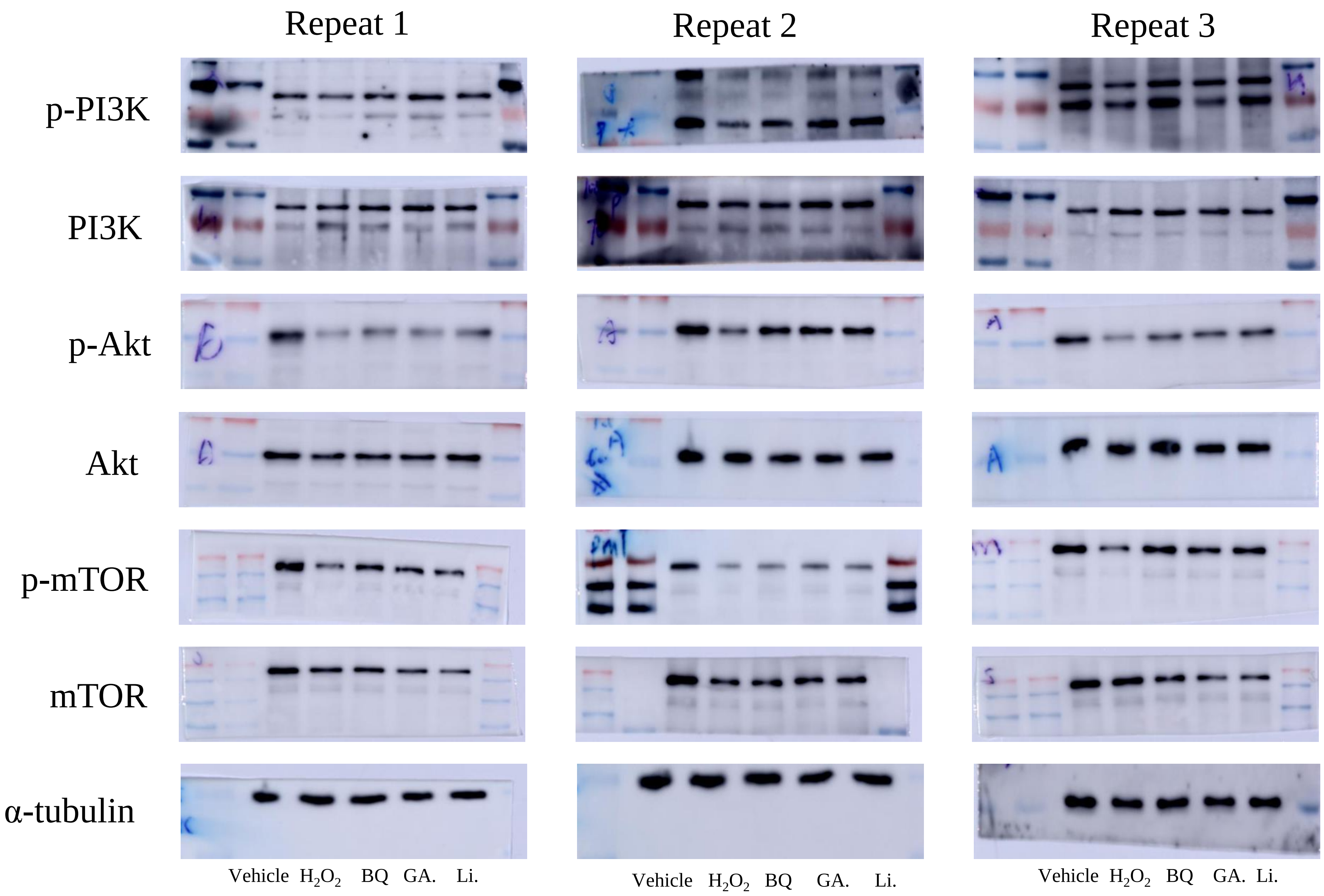

Supplementary material 5:

The whole images of the original western blots (For Figure 5J).

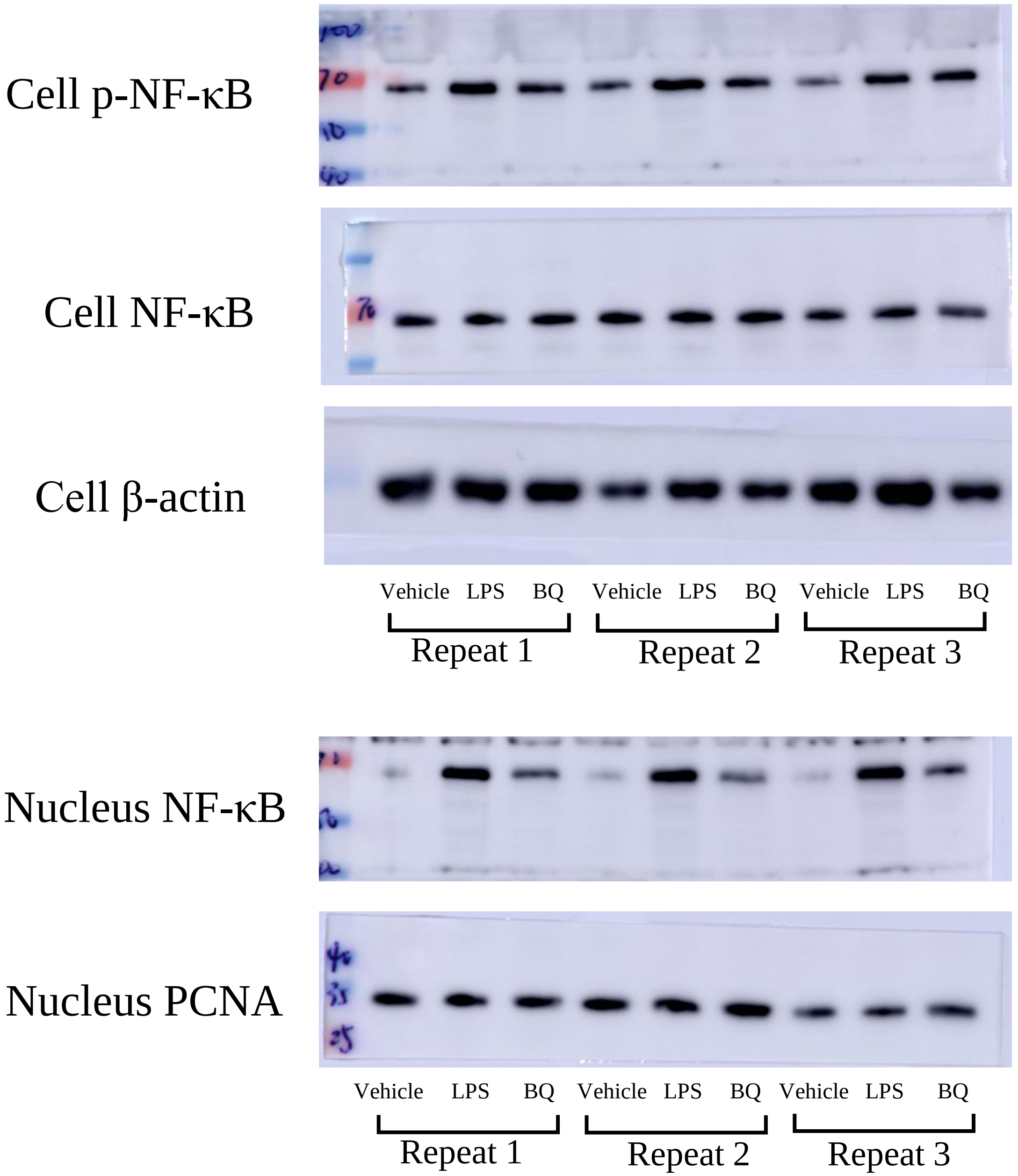

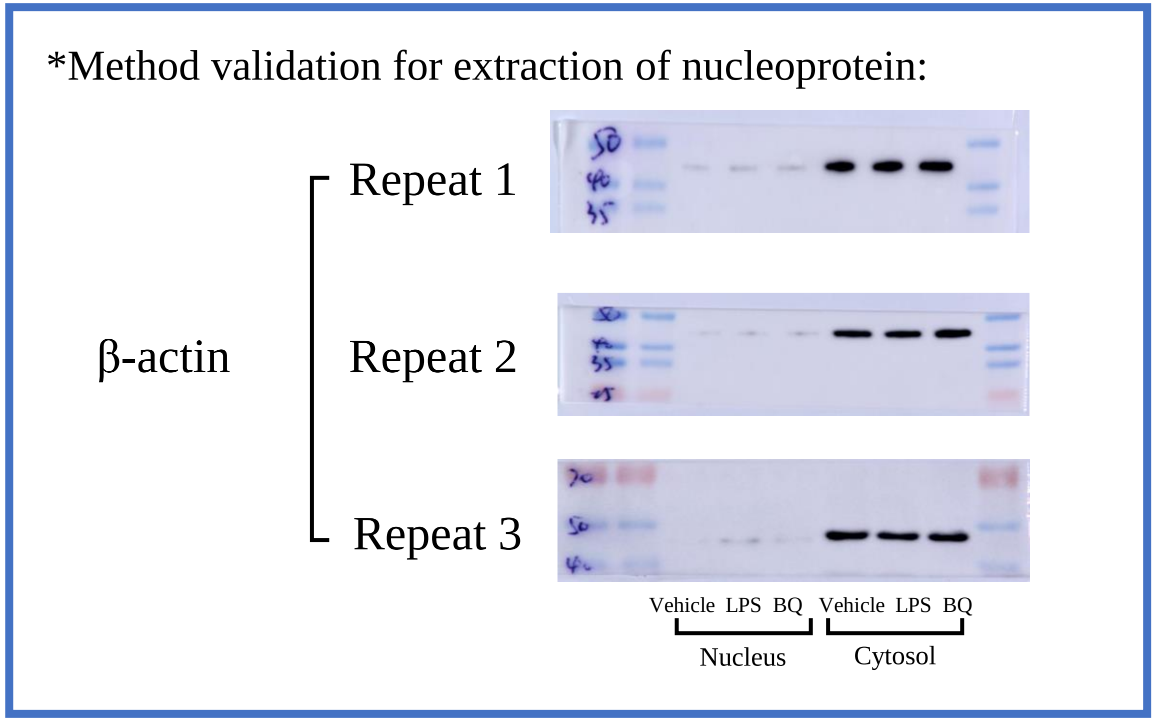

Supplement: Supplementary file 2 [file DataSheet1.PDF]
